# Evaluation of commercial point-of-care glucometers for detection and monitoring of neonatal hypoglycemia in resource-constrained settings

**DOI:** 10.1186/s12887-025-05934-9

**Published:** 2025-08-16

**Authors:** Meaghan Bond, Elizabeth Asma, Joseph Peterson, Lauren Oparah, Millicent Alooh, Danica Kumara, Z. Maria Oden, Chinyere Ezeaka, Elizabeth Molyneux, Rebecca Richards-Kortum

**Affiliations:** 1 Rice360 Institute for Global Health Technologies, Rice University, 6100 Main, Houston, TX 6100 USA; 2 San Francisco Bay Area, CA USA; 3 Department of Paediatrics, College of Medicine, University of Lagos, Lagos, Nigeria; 4 Department of Paediatrics, Kamuzu University of Health Sciences, Blantyre, Malawi

**Keywords:** Glucometer, Glucose, Neonate, Hypoglycemia, Pediatrics, Low-resource setting

## Abstract

**Background:**

Accurate identification and treatment of hypoglycemia or low blood glucose is important for neonates, especially premature, small for gestational age, and intra-uterine growth restricted (“small and sick”) newborns. Existing point-of-care (POC) adult glucometers, intended for home management of adults or children with diabetes, are widely available and less costly than machines designed and validated for neonates, but they must be validated at the lower glucose levels relevant for neonates and at appropriate hematocrit levels before being used for neonates.

**Methods:**

We simulated neonatal blood by altering hematocrit (to 18%, 40%, and 55%) and glucose concentration (to 40 mg/dL and 150 mg/dL) of normal adult blood. We then used 11 POC glucometers to measure glucose levels in these samples and compared results to values measured with a clinical chemistry analyzer (YSI 2300).

**Results:**

We report mean percent bias, mean absolute relative difference, and standard deviation for each tested condition and glucometer. We ranked the tested glucometers based on a combination of accuracy and precision for measuring neonatal glucose levels and consumable cost. From best-performing to worst, they are Group 1: StatStrip and StatStrip Xpress 2; Group 2: AccuChek Guide, AccuChek GuideMe, AccuChek Instant, and AccuChek Performa; Group 3: AccuChek Active, GlucoNavii; Group 4: HemoCue RT, Nipro Premier, and OneTouchUltra 2.

**Conclusions:**

This paper describes a simple, laboratory-based method to test glucometers across a wide range of hematocrit values and glucose concentrations and contributes up-to-date testing data on currently available POC glucometers at ranges relevant to neonatal use.

**Supplementary Information:**

The online version contains supplementary material available at 10.1186/s12887-025-05934-9.

## Background

Accurate identification and treatment of hypoglycemia (low blood glucose) is important for neonates, especially premature, small for gestational age, and intra-uterine growth restricted (“small and sick”) newborns [1, 2]. Sharma et al. recommend that glucose levels for preterm babies be checked within 4 h of birth and within 20 min of interventions, necessitating access to glucose diagnostics [1]. However, in many settings with high preterm birth rates, neonatal glucometers are expensive or unavailable [2, 3].

Existing point-of-care (POC) adult glucometers, intended for home management of adults or children with diabetes, are widely available and less costly than machines designed and validated for neonates. Validation of POC adult glucometers for neonatal use is important because the clinically important range of neonatal glucose concentration is much lower (approximately 25–79 mg/dL) than that of a diabetic adult outpatient (70–100 mg/dL) [3, 4].

Furthermore, neonates also have higher hematocrits than adults.^5^ Adult hematocrits typically range from 38–45% [5], whereas a newborn hematocrit is higher, ranging from 40 to 64% for healthy term neonates [6]. Small and sick newborns can also suffer from anemia due to younger gestational age or illness [7], so glucometers must also be validated across a wide range of hematocrits: a recent study in Malawi saw hematocrits as low as 18% [8]. Some POC adult glucometers have been shown to have lower accuracy for blood samples with either low or high hematocrit values [9, 10].

One possible reason for lower accuracy with low and high hematocrit values is many POC glucometers measure whole blood glucose and multiply by a correction factor (1.11) to report equivalent plasma glucose [11]. This correction factor assumes a normal adult hematocrit. Extreme values of hematocrit can affect the accuracy of this approach, and this error can be compounded with other difficulties, such as the increased blood viscosity at high hematocrits.

Thus, before using adult POC glucometers for neonates, their performance must be evaluated at glucose concentrations typical for neonates across a wide range of hematocrits. While other researchers have assessed the accuracy of using adult POC glucometers for neonatal blood [12–15], these analyses need to be frequently updated due to the constantly changing landscape of available POC glucometers. Additionally, while most evaluations use neonatal clinical blood samples tested on both a standard device and a POC glucometer, these evaluations are difficult to conduct due to the time to accrue patients, difficulty in acquiring samples near the high and low ends of the hematocrit and glucose ranges, and small volumes of blood samples that prevents repeated measurements and an assessment of precision.

To address these limitations, we report a simple method to simulate the hematocrit and glucose values of neonatal blood by altering hematocrit and glucose concentration of normal adult venous blood. We used these samples to evaluate the performance of currently available POC glucometers, comparing results to a clinical chemistry analyzer (YSI 2300). Based on these results, we recommend which currently available adult POC glucometers are best suited for use with neonatal samples when laboratory analyzers are not available.

## Methods

We simulated neonatal blood by altering hematocrit (to 18%, 40%, and 55%) and glucose concentration (to 40 mg/dL and 150 mg/dL) of normal adult blood. We then used POC glucometers to measure glucose levels in these samples and compared results to values measured with a clinical chemistry analyzer (YSI 2300).

### Choice of glucometers

UNICEF and NEST360 developed Target Product Profiles (TPP) for newborn care in low-resource settings [3]. No POC glucometers currently available meet all performance or cost metrics in the TPP. Glucometers with stringent regulatory approval for hospital use with newborns are often 20-fold higher in cost than specified by the TPP. Some glucometers that meet the cost specification of the TPP do not have regulatory approval for use with newborns and are only approved for use with adults.

We evaluated 11 models of glucometers; two models indicated for use with neonatal blood, four models with conditional statements regarding use with neonates and five models not indicated for use with newborns. Of the 11 glucometer models, eight devices met TPP specifications for instrument pricing (<$30 ex-works instrument price) while three indicated for use in neonates exceeded TPP specifications for instrument pricing by nearly 20-fold. The TPP specifies a minimal target of $0.20 per-test for consumables and an optimal target of $0.05. Of the five devices with quotes available from African distributors, three exceed the TPP’s minimal target by two- to three-fold (StatStrip, StatStrip Xpress 2, and HemoCue RT), and two meet the TPP (Accu-Chek Instant and Accu-Chek Active).

**Fig. 1:**
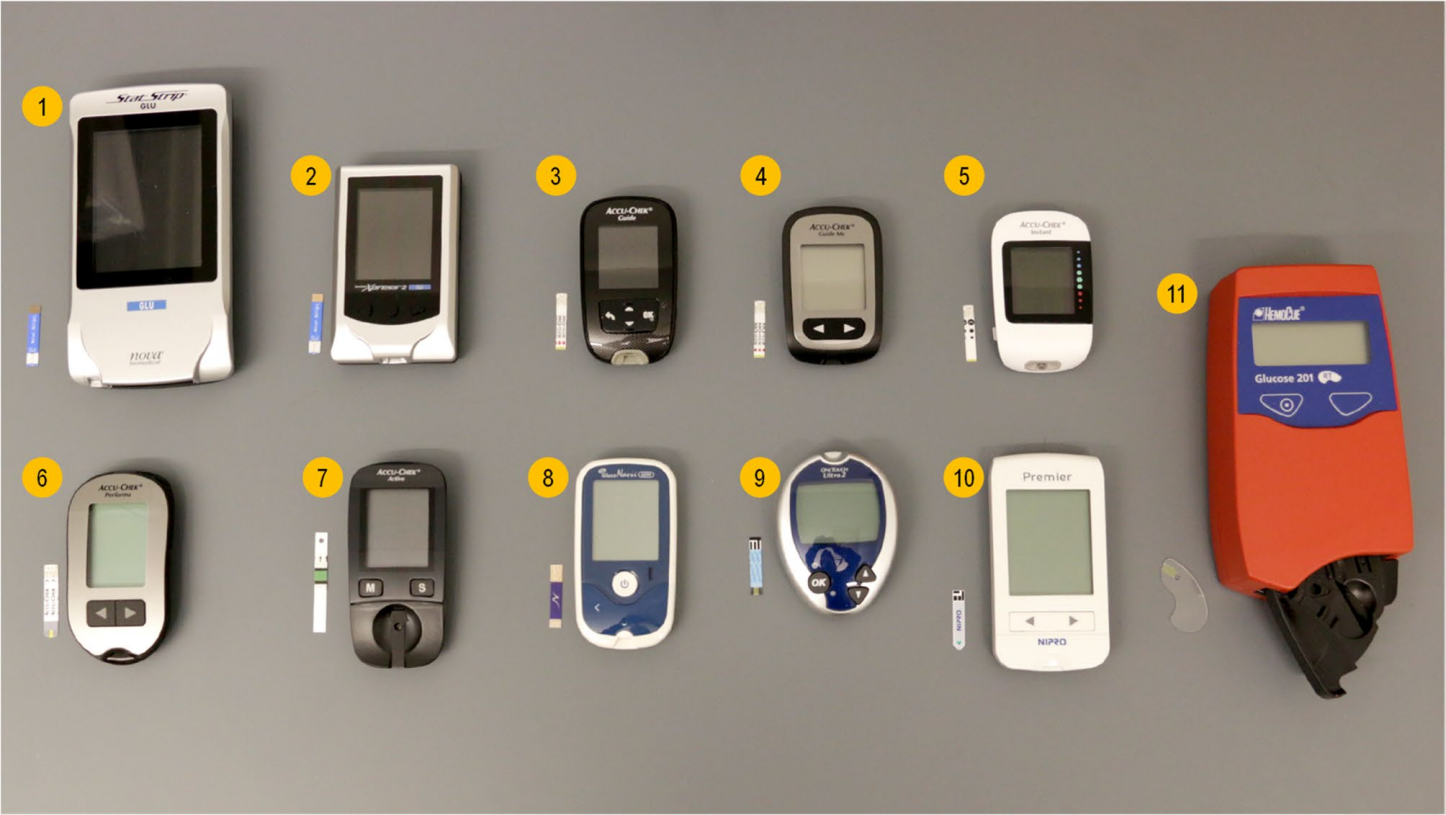
Photographs of tested glucometers and their test strips (1) Nova Biomedical StatStrip, (2) Nova Biomedical StatStripXpress2, (3) Accu-Chek Guide, (4) Accu-Chek Guide Me, (5) Accu-Chek Instant, (6) Accu-Chek Performa, (7) Accu-Chek Active, (8) GlucoNavii GDH, (9) OneTouch Ultra 2, (10) Nipro Premier, (11) HemoCue Glucose 201 RT

The devices and their consumable strips or cuvette are pictured in Fig. 1. Information about the glucometers according to the manufacturer’s product insert is shown in Table 1.

**Table 1:** Characteristics of tested glucometers

Manufacturer	Device	Technology^a^	Sample Volume	Intended Hct Range	Intended Glucose Range^b^	Indicated for use with neonatal blood?
Nova Biomedical	StatStrip	Glucose oxidase	1.2 μL	20-65%	10-600 mg/dL 0.6–33.3 mmol/L	Yes
Nova Biomedical	StatStrip Xpress 2	Glucose oxidase	1.2 μL	20-65%	10-600 mg/dL 0.6–33.3 mmol/L	Yes
Roche Diabetes Care	Accu-Chek Guide	FAD-GDH	0.6 μL	10-65%	20-600 mg/dL 1.1–33.3 mmol/L	No
Roche Diabetes Care	Accu-Chek GuideMe	FAD-GDH	0.6 μL	10-65%	20-600 mg/dL 1.1–33.3 mmol/L	No
Roche Diabetes Care	Accu-Chek Instant	GDH-FAD	0.6 μL	10-65%	10-600 mg/dL 0.6–33.3 mmol/L	Healthcare professional use
Roche Diabetes Care	Accu-Chek Performa	Mutant Q-GDH	0.6 μL	10-65%	10-600 mg/dL 0.6–33.3 mmol/L	Healthcare professional use
Roche Diabetes Care	Accu-Chek Active	Mutant Q-GDH, optical	1-2 μL	20-55% (in reader) 20-70% (applied outside of reader)	10-600 mg/dL 0.6–33.3 mmol/L	No
SD Biosensor	GlucoNavii	GDH	0.5 μL	0-70%	10-600 mg/dL 0.6–33.3 mmol/L	Conflicting statements in IFU^c^
LifeScan	OneTouchUltra 2	Glucose oxidase	1 μL	30-55%	20-600 mg/dL 1.1–33.3 mmol/L	No
I-SENS, Inc.	Nipro Premier	GDH-FAD	0.4 μL	15-65%	20-600 mg/dL 1.1-33.3 mmol/L	No
HemoCue	Glucose 201 RT	GDH, optical	5 μL	20-70%	0-500 mg/dL 0–27.8 mmol/L	Yes, with conditions^d^

Characteristics of the glucometers tested in this study, according to each manufacturer

^a^FAD-GDH: flavin-dependent glucose dehydrogenase

^b^Unit conversion: Glucose mmol/L * 18 = Glucose mg/dL

^c^“This can be used to test neonates for professional use… This system should not be used for…testing newborns”

^d^ Instructions for use exclude use for “critically ill neonates in neonatal intensive care settings”

### Sample preparation

Venous blood was drawn from normal, healthy adult volunteers under a protocol approved by the Rice University IRB. Written informed consent was obtained from all participants. Clinical trial number: not applicable. Blood was collected into lithium heparin vacutainers; this anticoagulant was approved for use with all tested glucometers. We then prepared samples with varying target hematocrits (18%, 40%, 55%) and target glucose concentrations (40 mg/dL, 150 mg/dL).

Immediately following collection, the starting glucose and hematocrit were measured for each sample. Target glucose concentrations were 40 mg/dL and 150 mg/dL, which represent hypo- and hyperglycemic values for newborns, respectively [4, 16]. When the sample required increased glucose, glucose (D-Glucose, NIST917c) at 50 mg/mL in 1x phosphate buffered saline (PBS) was added, and the sample was mixed thoroughly. The sample was allowed to sit for approximately 15 min to allow glucose to equilibrate between red blood cells and plasma. To decrease glucose, blood tubes were placed in a water bath at 37 °C on a stir plate and allowed to sit for up to several hours until the glucose had reached the desired concentration due to blood cells continuing to consume glucose. In cases where the sample glucose concentration dropped below the desired value, concentrated glucose was added as described above. These methods of increasing and decreasing glucose are in accordance with BS EN ISO 15197:2015, Sect. 6.1.2 [17].

After glucose concentration was adjusted, hematocrit was adjusted by centrifuging the blood (5 min, 1500 g) and removing or adding plasma with a matching glucose concentration. Target hematocrits were 18%, 40%, and 55%. The final hematocrit was then measured on a Coulter Ac∙T diff2 hematology analyzer (Beckman Coulter Diagnostics).

Because glycolysis continues in living red blood cells ex vivo, all samples were placed on ice to reduce glycolysis when the desired plasma glucose concentration was achieved.

### Device evaluation

For each experiment, glucometer unit number, consumable lot number, and consumable expiration date were noted. For those devices with a control solution accessible to the researchers (8/11), quality control was performed according to the manufacturer’s instructions. Only devices that passed were assessed. The remaining three devices did not have a control solution accessible to the researchers in the U.S. during testing (Accu-Chek Performa, Accu-Chek Active, Nipro Premier); these devices were used without performing a quality control check.

To prepare the YSI 2300 Stat Plus analyzer, we performed linearity tests and compared to limits set by the manufacturer. Two glucose solutions of known concentration (YSI2801 50 mg/dL, YSI2714 100 mg/dL) were measured on the YSI (YSI was set to not correct for hematocrit in this measurement). The YSI specifies linearity to be better than +/−5%; if measured values were within 5% of the stated value, the experiment proceeded. During Run mode, the YSI self-calibrates every 15 min or every 5 samples; it will not allow samples to be measured until it passes the self-calibration test. Glucose measurement membranes were replaced on a schedule recommended by the manufacturer. After measuring linearity samples, the YSI was then reset to measure blood in hematocrit correction mode, in which the user enters the measured hematocrit, and the device measures the whole blood sample and reports the corrected plasma glucose value.

We measured glucose concentration for samples with each combination of glucose concentration (40 mg/dL, 150 mg/dL) and hematocrit (18%, 40%, 55%). Five replicates were measured per day for each combination. Before each replicate, the blood sample was mixed gently by inversion or pipette.

A measurement was taken on the YSI, inputting sample hematocrit as measured by an Ac∙T diff2 hematology analyzer. Then, approximately 30 uL of the blood sample was pipetted onto Parafilm. The volume pipetted varied slightly depending on the number of devices to be evaluated in the experiment; the volume was determined so that there would be blood left in the drop after all devices had been evaluated.

Each glucometer was quickly loaded with strips, then blood was provided to each device and the glucose result recorded. For each of the five replicates per sample, the order of devices was reversed. Samples were kept on ice between replicates to reduce ongoing glycolysis. Measuring 5 replicates of a given sample on all of the devices took approximately 10–15 min.

This process was repeated with multiple blood donors across multiple days. Some models had multiple units examined, and some had multiple strip lots represented. A summary can be found in Table 2.

**Table 2:** Description of testing parameters by model of glucometer

Device	Results reported in	# Units Tested	# Days Tested	# Valid samples	# Errors (%)
StatStrip	mg/dL	2	16	282	0 (0%)
StatStrip Xpress 2	mg/dL	2	16	285	0 (0%)
Accu-Chek Guide	mg/dL	2	6	240	0 (0%)
Accu-Chek GuideMe	mg/dL	2	6	240	0 (0%)
Accu-Chek Instant	mmol/L	2	9	239	0 (0%)
Accu-Chek Performa	mg/dL	1	4	60	0 (0%)
Accu-Chek Active	mg/dL	2	6	240	0 (0%)
GlucoNavii	mg/dL	2	16	310	0 (0%)
OneTouchUltra 2	mg/dL	2	13	304	0 (0%)
Nipro Premier	mmol/L	2	7	106	4 (3.6%)
HemoCue Glucose 201 RT	mg/dL	2	9	262	9 (3.3%)

Testing parameters, such as the number of units used and the number of days tests were conducted, varied by model of glucometer and are summarized in this table

Some glucometers reported results in mmol/L (see Table 2). These results were converted to mg/dL according to the formula:

Glucose mmol/L * 18 = Glucose mg/dL.

### Analysis

When a device reported an error that was caused by the user, e.g. not fully inserting the strip or not applying enough blood, the measurement was either repeated or the replicate was dropped. All other errors reported by the device, such as low glucose value or faulty strip, were recorded and counted.

It was not possible to create samples with precisely the same hematocrit and glucose concentration across multiple days of experiments. Therefore, for each target group of glucose concentration and hematocrit value, we assessed the percent deviation between the value measured with the test glucometer and the YSI reference standard. For each condition on a given day, the average of the five YSI measurements was considered the true value. We then computed the percent deviation of each test glucometer data point from that average and compared it to established accuracy guidelines. We considered two accuracy guidelines. First, we analyzed whether a test device reported results within ± 8% of the true value based on the minimal accuracy requirement from the UNICEF-NEST360 Glucometer TPP [3] and the proposed Clinical Laboratory Improvement Amendments (CLIA) acceptance limit [18]. Second, we analyzed whether measured glucose values for a test device were within ± 15% of the true value, based on ISO 15197:2015 limits for glucose values ≥ 100 mg/dL [17]. We chose to use these percentage limits for both high and low glucose values to enable easy comparison across devices.

### Role of the funding source

The funders were not involved in study design; collection, analysis, or interpretation of data; in the writing of the report; or in the decision to submit the paper for publication.

## Results

Table 2 summarizes the number of samples and units evaluated for each device. Due to supply chain challenges, not every model was tested on every experiment day. All devices were tested with at least two strip lots except for Accu-Chek Performa, for which only one lot was accessible. All tested devices had some error detection capability; however, across all our testing only two models reported errors: Nipro premier (*n* = 4/110, “lo” error for low glucose concentration) and HemoCue (*n* = 9/271, all E70-E71, “the cuvette is faulty or the sample might be grossly lipemic.”)

**Fig. 2:**
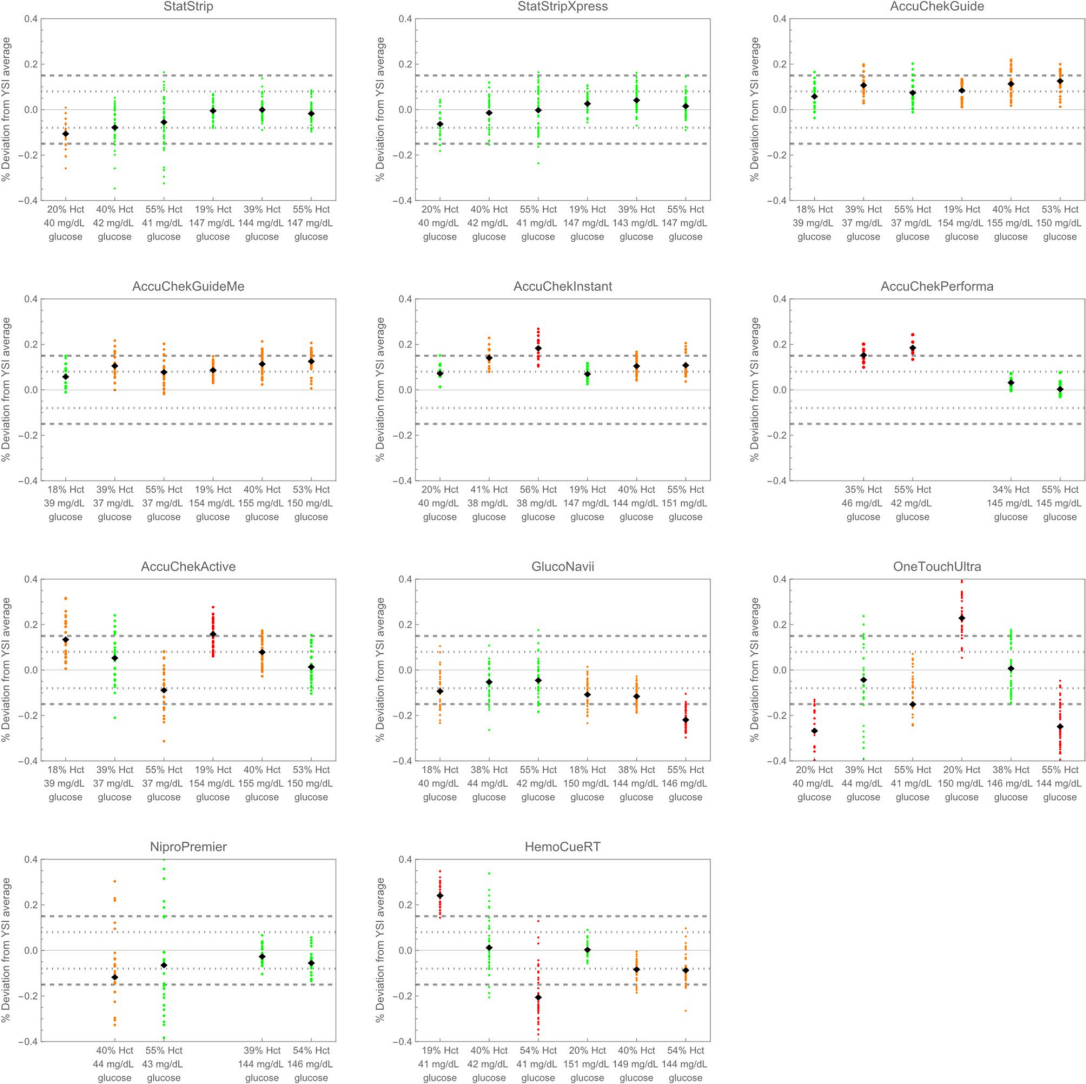
Accuracy summary of tested glucometers. Horizontal lines represent ± 8% and ± 15% deviation from the YSI average, respectively. Conditions are described on the x-axis with the average hematocrit and glucose concentration for the displayed data points. Groups of data are colored based on their mean percent bias from the YSI: green for ≤ 8% deviation, orange for > 8% and ≤ 15%, red for > 15%

Because glycolysis continues in ex vivo samples, there was some concern that the concentration of glucose would decrease during the measurement of 5 replicates. We assessed this by plotting the YSI measurements across the 5 replicates; no relevant changes were observed (Supplemental Figure 2). Figure 2 compares the performance of each model of glucometer to the YSI stratified by hematocrit value and glucose concentration. Data from all units of a particular model are combined in Fig. 2; Supplemental Fig. 1 shows results for each test unit. The horizontal dashed lines represent two accuracy standards: ±8% of the YSI average, from the UNICEF-NEST360 Glucometer TPP [3] and the proposed CLIA acceptance limit [18], and ± 15% of the YSI average, based on ISO 15197:2015 [17]. Groups of data points are colored based on the mean percent bias of all data points from the YSI average:

$$\:Mean\:Percent\:Bias=Mean\:\left(\frac{meter\:value-average\:YSI\:value}{average\:YSI\:value}*100\right)$$

A mean percent bias within ± 8% is colored green, between ± 8% and ± 15% is orange, and greater than ± 15% is red.

**Fig. 3:**
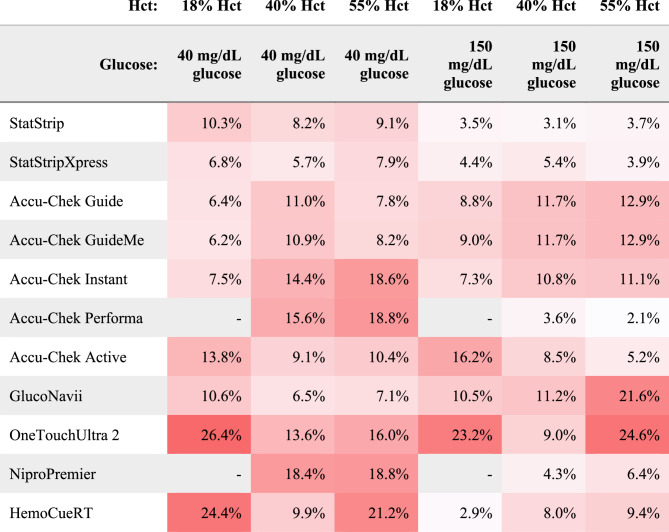
Mean absolute relative difference from the YSI for each model, calculated according to Ekhlaspour (22)

Figure 3 reports the mean absolute relative difference (MARD, calculated according to Ekhlaspour^15^) for each model and condition.

$$\:MARD=Mean\left(\frac{\left|meter\:value-average\:YSI\:value\right|}{average\:YSI\:Value}*100\right)$$

Mean percent bias and standard deviation are reported in Supplemental Table 1. Supplemental Table 2 uses the mean percent bias, standard deviation, and nominal glucose value to report the 95% limits of agreement for each device, the range within which each device would be expected to report 95% of glucose values for a given glucose concentration and hematocrit level.

NovaBiomedical’s StatStrip and StatStripXpress 2 showed the best accuracy, with a mean percent bias within ± 8% of the reference standard across all but one tested condition (StatStrip had a mean percent bias of −10.3% at the lowest Hct and glucose). At low glucose, there was a weak association of mean percent bias decreasing with decreasing hematocrit. There was a decreased precision when measuring low glucose concentrations.

We evaluated five models branded Accu-Chek: Guide, GuideMe, Instant, Performa, and Active. The Performa model had limited strip availability during our testing, so it was only evaluated for samples with 40% and 55% Hct. The Accu-Check Guide and Guide me all showed a mean percent bias below ± 15% and high precision with little bias across the range of tested hematocrits. The Accu-Check Instant and Performa showed similarly high precision, but 1–2 tested conditions outside the ± 15% mean percent bias guidelines. All tested conditions were within ± 20% mean percent bias; ±20% guidelines were recommended in the older 2003 edition of the ISO standard [19]. Performance was similar for models where multiple units were tested (Supplemental Fig. 1). The Active was the least precise of the Accu-Chek models evaluated in this paper. It showed a slight negative bias with increasing hematocrit.

GlucoNavii slightly underestimated glucose values across all conditions, with mean percent bias ranging from − 5.0% to −22%. It had greater accuracy with lower glucose levels and did not show a dependence on hematocrit at low glucose. Both units tested underestimated glucose levels at high hematocrit and high glucose. GlucoNavii showed a higher imprecision than most of the Accu-Chek models.

OneTouchUltra 2 showed poor accuracy at the extremes of hematocrit and poor precision across all conditions tested.

NiPro Premier demonstrated good accuracy, but very poor precision at low glucose levels (standard deviation 19.6% and 21.5%). Because of this high imprecision and difficulty acquiring strips, it was not evaluated at the lowest hematocrit levels.

Glucose values measured with the HemoCue Glucose 201 RT differed substantially (> 20%) from the reference standard at low glucose and either high or low hematocrit values. Results at low glucose values were highly variable. Both units evaluated showed similar performance issues (Supplemental Fig. 1). At elevated glucose concentrations, the HemoCue Glucose 201 RT yielded more accurate and precise results. Nine (3.3%) of the measurements resulted in error codes necessitating a new cuvette and blood sample.

**Fig. 4:**
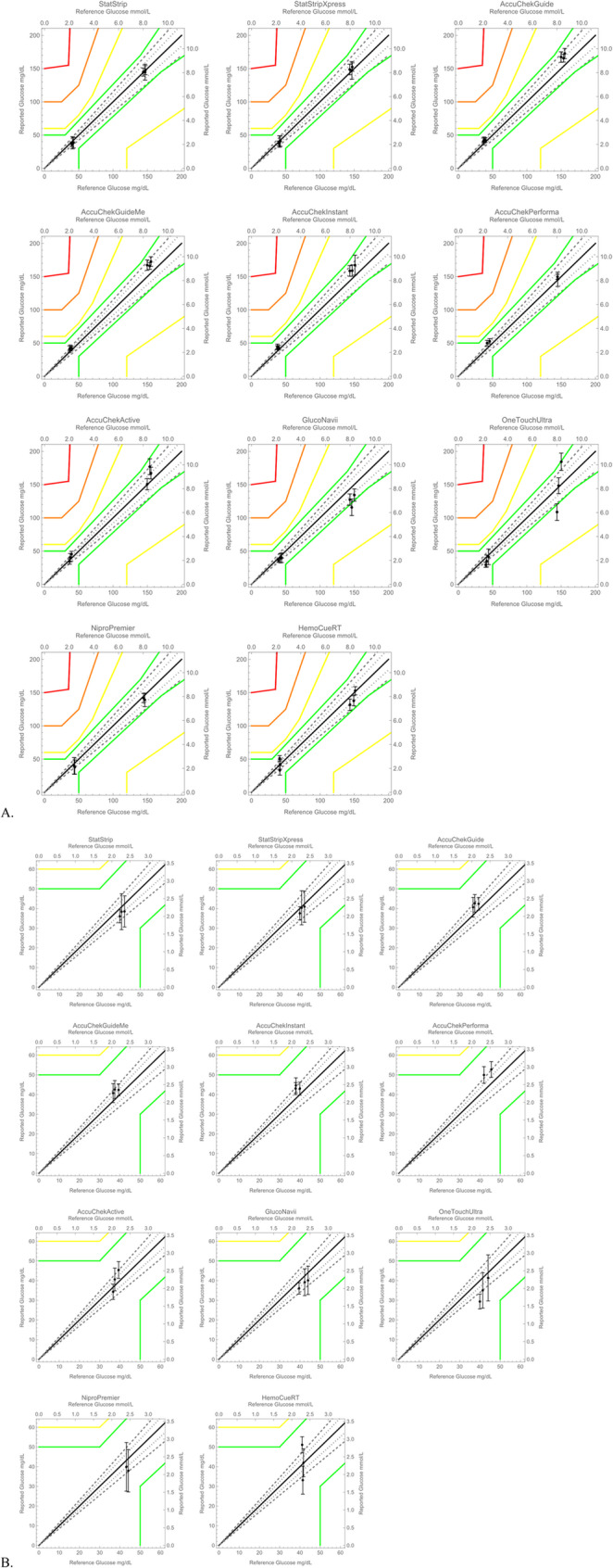
Glucometer accuracy summary on Parkes error grid. Average ± 1 standard deviation of test device glucose plotted on a Parkes error grid for Type 1 diabetes, which is used for regulatory, as described by Pfützner et al. [20]. Between the green and yellow lines is zone B, “altered clinical results, little or no effect on clinical outcome;” between yellow and orange is zone C, “altered clinical action, likely to affect clinical outcome;” between orange and red is zone D, “altered clinical action, could have significant clinical risk;” and outside red is zone E, “altered clinical action, could have dangerous consequences.” Solid black line is line of equality; dotted gray line is ± 8%; dashed gray line is ± 15%. Each data point represents the average of a given hematocrit and glucose. **A** Plot range 0–200 mg/dL, **B** zoomed in plots to show the range 0–60 mg/dL

These results demonstrate the performance of various glucometers at glucose and hematocrit ranges relevant for neonates. In order to determine which are appropriate for clinical use, we also plotted the data on a Parkes error grid (Fig. 4). This error grid was described by Pfützner et al. for Type 1 diabetes and is used for regulatory purposes [20]. Klonoff et al. recently published a revised Diabetes Technology Society error grid updating the Parkes and Surveillance error grids; the new Diabetes Technology Society error grid has a very similar Zone A/Zone B boundary to the Parkes error grid shown here, and both grids would result in the same conclusions [21]. No error grids have been specifically developed for neonates, and thus these error grids may not correctly estimate the accuracy required for treating this vulnerable population.

In the error grids in Fig. 4, all devices have all average values at low glucose levels within Zone A (“no effect on clinical action”). GlucoNavii and OneTouchUltra 2 each have one average at high glucose values in Zone B (“altered clinical results, little or no effect on clinical outcome”).

## Discussion & conclusions

NovaBiomedical’s StatStrip and StatStripXpress 2 are indicated for use in neonates and critically ill patients (https://novabiomedical.com/statstrip-glu/index.php). They also have the highest cost. All points are within zone A on the error grid. This performance agrees with that shown by Ehklaspour et al., who reported an overall MARD (Mean Absolute Relative Difference) of 6.3% for StatStripXpress [22]; in our experiments, the MARD ranged from 3.9 to 7.9% across the tested conditions (Fig. 3). Precision is lower at low glucose levels; precision is important in neonatal care in order to detect trends in patient glucose concentration.

The Accu-Chek Instant and Performa are indicated for use with neonates when used by healthcare professionals; the Guide, GuideMe, and Active are not indicated for use with neonates. However, their good accuracy, high precision, and low cost make these units an attractive option for neonatal use in low-resource settings. All data points are within zone A on the error grid. The Active model uses an optical measurement technique, in contrast to the amperometric method used by all other Accu-Chek models evaluated in this paper. In a clinical study in Cameroon on adults, the Accu-Chek Active had a mean difference from the reference of 17.4% [23], comparable to our MARDs of 5.2 − 16.2% (Fig. 3).

The instructions for use for GlucoNavii have conflicting statements about indications for neonatal use; its good accuracy at low glucose suggests it could be an acceptable choice for neonatal units in low-resource settings. However, the readings underestimate high glucose levels, and one data point is in zone B on the error grid.

For OneTouchUltra 2, Ekhlaspour observed high error at low glucose: MARD of 30.3% for glucose < 70 mg/dL [22]; in our experiment, the OneTouchUltra 2 had a MARD of 26.4%, 13.6%, and 16.0% at glucose levels approximately 40 mg/dL for low, medium, and high hematocrits, respectively (Fig. 3). At high glucose and the two extremes of hematocrit, the data points are in zone B of the error grid. In a clinical study in Cameroon on adults, OneTouchUltra 2 had a mean difference from the reference of 12%, though this study compared capillary blood samples on the OneTouchUltra 2 to venous samples on a laboratory analyzer [23]. The manufacturer states it is not indicated for neonatal use.

NiPro states this model is not indicated for neonatal use, and this is appropriate based on its low precision for samples with low glucose values.

Despite indications for use with neonates, the poor accuracy observed for HemoCue Glucose 201 RT does not support safe use in neonates. Additionally, the high cost of HemoCue cuvettes will limit its use in low-resource settings. Ekhlaspour et al. likewise reported poor performance of HemoCue at low glucose values: a 19.9% MARD ± 19.4 SD from the YSI for glucose below 70 mg/dL [22]; in our experiments, HemoCue showed a MARD of 24.4%, 9.9%, and 21.2% at glucose near 40 mg/dL for low, medium and high hematocrits, respectively (Fig. 3). Of note, HemoCue uses an optical detection method, whereas most other meters use an amperometric detection method.

We note that the lowest tested Hct in this study (18%) is below the intended Hct range of some tested glucometers, as detailed in Table 1: StatStrip (lower limit of 20% Hct), StatStrip Xpress 2 (20%), Accu-Chek Active (20%), OneTouchUltra 2 (30%), and HemoCue Glucose 201 (20%). Three devices (Accu-Chek Guide, Accu-Chek GuideMe, and OneTouchUltra 2) do not allow the use of venous blood.

**Fig. 5:**
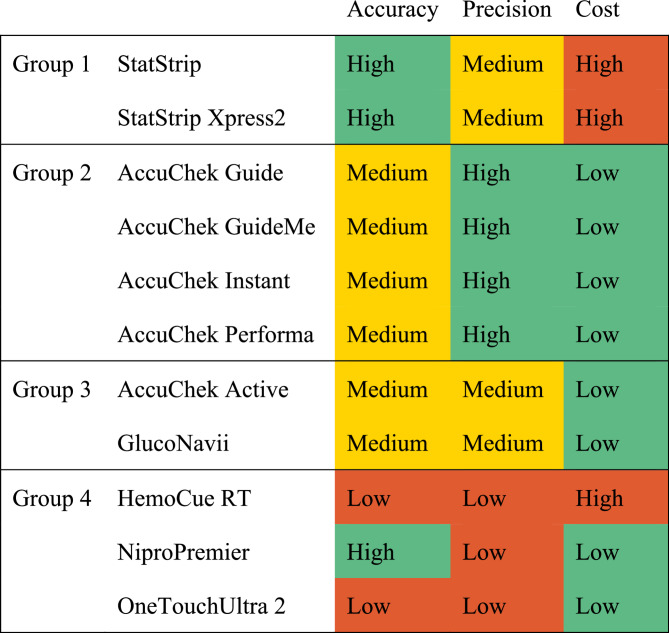
Ranking of tested glucometers from best performing (Group 1) to worst (Group 4) for neonatal glucose measurements

Based on the data in Fig. 2, we ranked the tested devices for use in neonatal glucose measurements (Fig. 5). Glucometers in Group 1 have good performance, but their high cost may limit availability in low-resource settings. Glucometers in Group 2 have the next best performance and may be more widely available in low-resource settings. Group 3 glucometers have some shortcomings in accuracy or precision under some conditions of glucose levels or hematocrit; clinicians should be aware of these limitations when using glucometers from this group. Glucometers in Group 4 have serious accuracy or precision errors in conditions important for neonatal use and are not recommended for this use case. Clinicians and hospital purchasing agents should use this information to weigh the device performance, cost, and availability in their region to determine an appropriate glucometer for neonatal hospital use.

We note a few limitations in our study. While our test parameters span a range of representative hematocrits and glucose levels found in neonates, further testing could explore the extremes of this range, such as very high hematocrit (65%) and very low glucose concentrations. Our method mimics the parameters of neonatal blood that are most relevant to blood glucose measurement (glucose and hematocrit), but does not replicate all differences between adult and neonatal blood, such as a larger mean cell volume (MCV), higher RBC distribution width (RDW), differences in RBC metabolism, or higher WBC count in neonates [7]. This study was necessarily conducted on venous blood, but most neonatal measurements in clinical practice will be conducted on capillary blood. Storing the samples on ice between measurements may have led to a small degree of hemolysis; while the measurements on the YSI did not show any effect (Supplemental Figure 5) and no lysis was observed visually (e.g. plasma remained clear), the point of care glucometers could have been more strongly affected. We chose to evaluate a low, clinically relevant glucose threshold of 40 mg/dL; future studies could use these methods to evaluate other hypoglycemic thresholds [4].

In conclusion, this paper describes a simple, laboratory-based method to test glucometers across a wide range of hematocrit values and glucose concentrations and contributes up-to-date testing data on currently available POC glucometers at ranges relevant to neonatal use.

## Supplementary Information

Supplementary Material 1

## Data Availability

The laboratory dataset supporting the conclusions of this article is available from the corresponding author on reasonable request.

## Abbreviations

POC: Point-of-care
TPP: Target product profiles
PBS: Phosphate buffered saline
CLIA: Clinical laboratory improvement amendments
MARD: Mean absolute relative difference
