# Supplementary material for: Evaluation of commercial point-of-care glucometers for detection and monitoring of neonatal hypoglycemia in resource-constrained settings
Source: BMC Pediatr. 2025 Aug 16;25:624. doi: 10.1186/s12887-025-05934-9 (PMC12357331; doi:10.1186/s12887-025-05934-9)
Supplement: Supplementary file 1 — Supplementary Material 1 [file 12887_2025_5934_MOESM1_ESM.docx]

Evaluation of Commercial Point-of-Care Glucometers for Detection and Monitoring of Neonatal Hypoglycemia in Resource-Constrained Settings

Supplemental


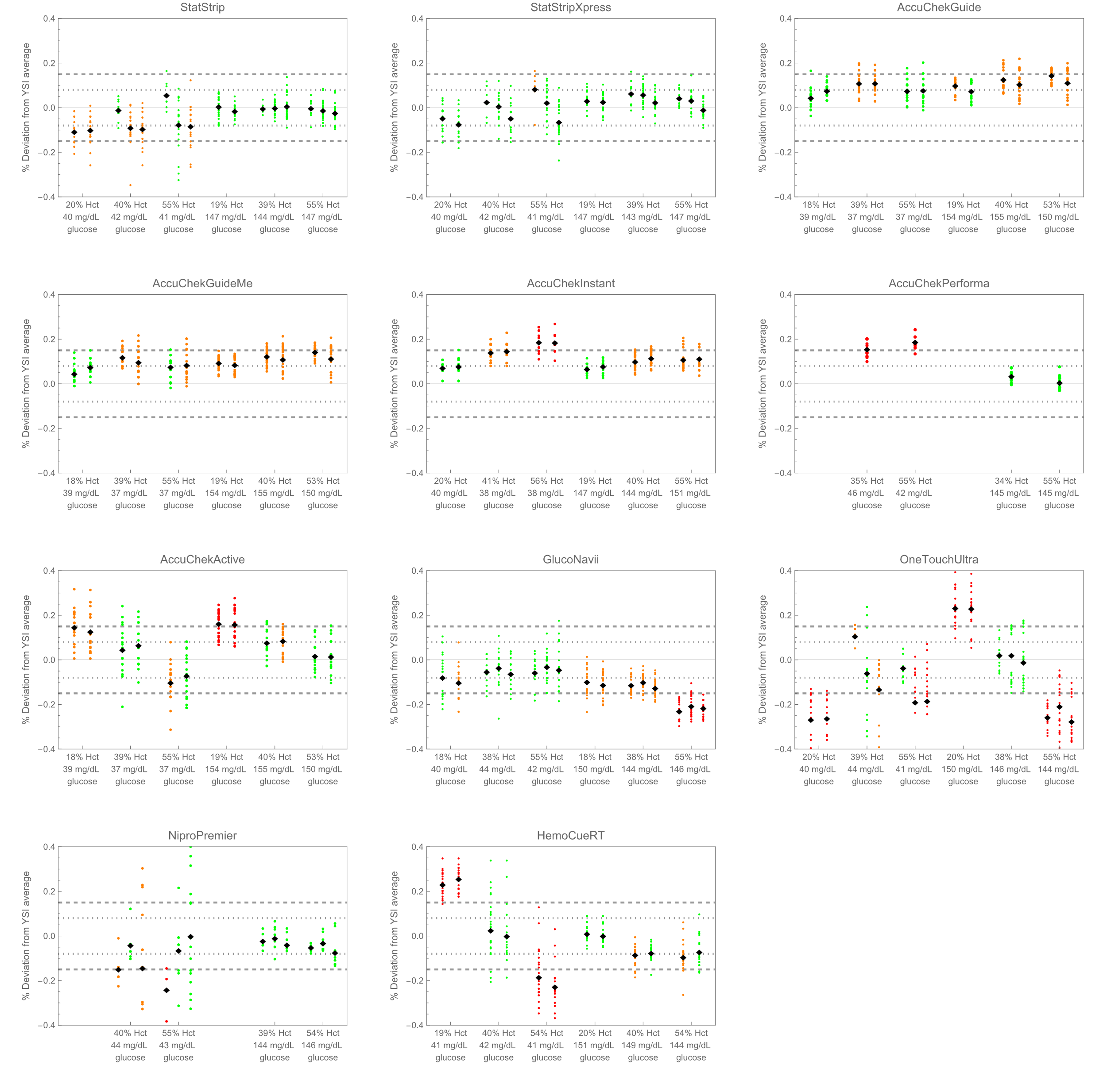


*Supplemental Figure 1.* Accuracy summary of tested glucometers, separated by individual unit. For conditions with three columns of data points (e.g. StatStrip at 40% Hct, 42 mg/dL glucose), the first column represents “Unit number not recorded”, the second column “Unit #1”, and the third “Unit #2”. Horizontal lines represent ±8% and ±15% deviation from the YSI average, respectively. Conditions are described on the x-axis with the average hematocrit and glucose concentration for the displayed data points. Groups of data are colored based on their average deviation from the YSI: green for ≤ 8% deviation, orange for > 8% and ≤ 15%, red for > 15%.

1.
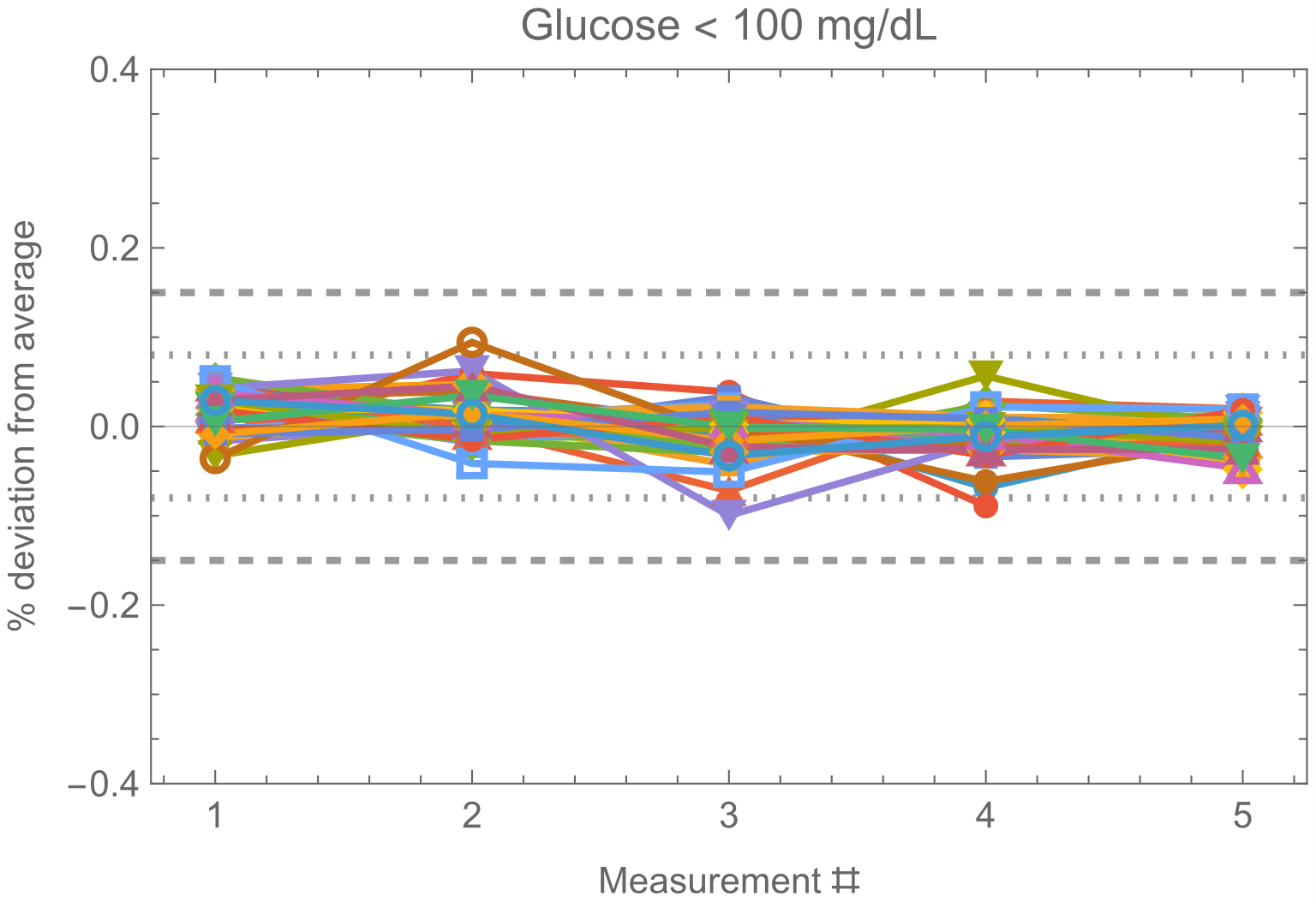

2.
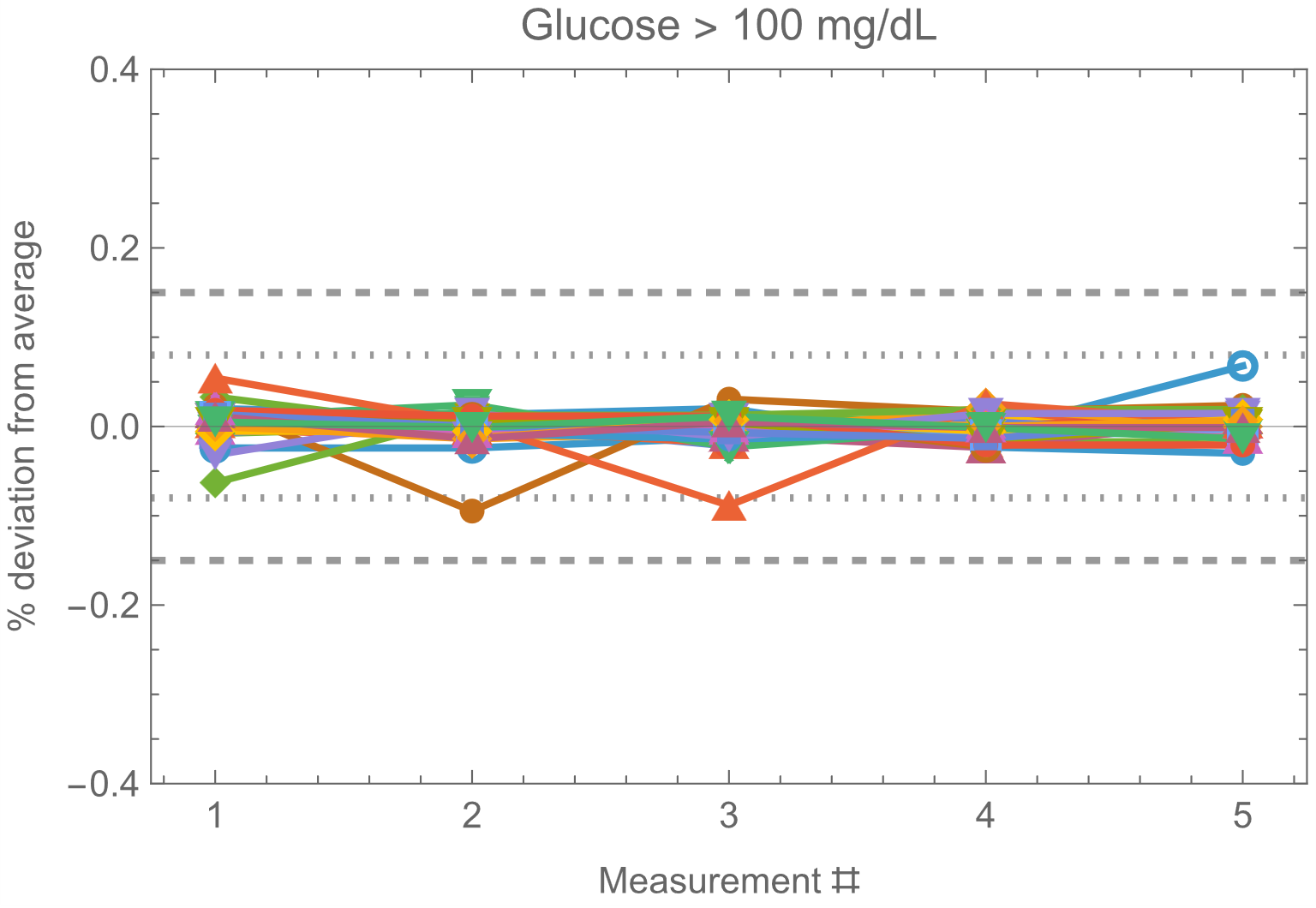


*Supplemental Figure 2.* Percent deviation of each YSI measurement from the average of all YSI measurements for that condition and day. The small changes indicate that there was not significant glycolysis during the measurement of 5 replicates. (A) Low glucose values, (B) high glucose values.

*Supplemental Table 1.* Top: Average percent deviation from the YSI for each model by Hct and glucose condition. Bottom: standard deviation of percent deviation from the YSI for each model.

|  | 18% Hct | 40% Hct | 55% Hct | 18% Hct | 40% Hct | 55% Hct |
| --- | --- | --- | --- | --- | --- | --- |
| Mean % Bias from YSI: | **40 mg/dL glucose** | **40 mg/dL glucose** | **40 mg/dL glucose** | **150 mg/dL glucose** | **150 mg/dL glucose** | **150 mg/dL glucose** |
| StatStrip | -10.3% | -7.5% | -5.2% | -0.2% | 0.3% | -1.4% |
| StatStripXpress 2 | -6.0% | -1.1% | 0.1% | 2.9% | 4.5% | 1.9% |
| Accu-Chek Guide | 6.1% | 11.0% | 7.7% | 8.8% | 11.7% | 12.9% |
| Accu-Chek GuideMe | 6.1% | 10.9% | 8.1% | 9.0% | 11.7% | 12.9% |
| Accu-Chek Instant | 7.5% | 14.4% | 18.6% | 7.3% | 10.8% | 11.1% |
| Accu-Chek Performa | **-** | 15.6% | 18.8% | - | 3.5% | 0.6% |
| Accu-Chek Active | 13.8% | 5.6% | -8.6% | 16.2% | 8.2% | 1.7% |
| GlucoNavii | -9.0% | -5.0% | -4.2% | -10.5% | -11.2% | -21.6% |
| OneTouchUltra 2 | -26.4% | -4.0% | -14.8% | 23.2% | 1.0% | -24.6% |
| NiproPremier | **-** | -11.4% | -6.1% | - | -2.3% | -5.2% |
| HemoCueRT | 24.4% | 1.6% | -20.3% | 0.6% | -8.0% | -8.4% |
|  |  |  |  |  |  |  |
|  |  |  |  |  |  |  |
| Standard Deviation: |  |  |  |  |  |  |
| StatStrip | 6.1% | 7.6% | 11.0% | 4.2% | 4.2% | 4.5% |
| StatStripXpress 2 | 6.2% | 6.9% | 9.3% | 4.3% | 5.1% | 4.5% |
| Accu-Chek Guide | 4.3% | 4.4% | 5.2% | 4.0% | 5.1% | 4.7% |
| Accu-Chek GuideMe | 4.2% | 5.4% | 5.7% | 3.5% | 4.8% | 4.5% |
| Accu-Chek Instant | 3.1% | 4.0% | 4.0% | 2.5% | 3.5% | 4.5% |
| Accu-Chek Performa | **-** | 3.5% | 2.9% | - | 2.4% | 2.8% |
| Accu-Chek Active | 8.6% | 10.0% | 9.2% | 6.1% | 5.6% | 6.7% |
| GlucoNavii | 8.8% | 6.9% | 8.0% | 6.2% | 4.1% | 4.2% |
| OneTouchUltra 2 | 9.4% | 16.7% | 15.8% | 8.2% | 10.4% | 9.3% |
| NiproPremier | **-** | 19.6% | 21.5% | - | 4.4% | 5.2% |
| HemoCueRT | 6.0% | 13.0% | 11.2% | 3.8% | 4.1% | 6.4% |

*Supplemental Table 2.* 95% limits of agreement in mg/dL for each model by hematocrit and glucose level. Limits of agreement calculated by (Mean % bias ± 2 * standard deviation + 1)*(Nominal glucose value). Nominal glucose values are 40 mg/dL or 150 mg/dL, shown at top of column.

| Hct | 18% | 40% | 55% | 18% | 40% | 55% |
| --- | --- | --- | --- | --- | --- | --- |
| Glucose | **40 mg/dL** | **40 mg/dL** | **40 mg/dL** | **150 mg/dL** | **150 mg/dL** | **150 mg/dL** |
| StatStrip | 31 - 41 | 31 - 43 | 29 - 47 | 137 - 162 | 138 - 163 | 134 - 161 |
| StatStripXpress 2 | 33 - 43 | 34 - 45 | 33 - 47 | 141 - 167 | 141 - 172 | 139 - 166 |
| Accu-Chek Guide | 39 - 46 | 41 - 48 | 39 - 47 | 151 - 175 | 152 - 183 | 155 - 183 |
| Accu-Chek GuideMe | 39 - 46 | 40 - 49 | 39 - 48 | 153 - 174 | 153 - 182 | 156 - 183 |
| Accu-Chek Instant | 41 - 45 | 43 - 49 | 44 - 51 | 153 - 168 | 156 - 177 | 153 - 180 |
| Accu-Chek Performa |  | 43 - 49 | 45 - 50 |  | 148 - 162 | 143 - 159 |
| Accu-Chek Active | 39 - 52 | 34 - 50 | 29 - 44 | 156 - 193 | 146 - 179 | 132 - 173 |
| GlucoNavii | 29 - 43 | 32 - 44 | 32 - 45 | 116 - 153 | 121 - 146 | 105 - 130 |
| OneTouchUltra 2 | 22 - 37 | 25 - 52 | 21 - 47 | 160 - 209 | 120 - 183 | 85 - 141 |
| NiproPremier |  | 20 - 51 | 20 - 55 |  | 133 - 160 | 127 - 158 |
| HemoCueRT | 45 - 55 | 30 - 51 | 23 - 41 | 140 - 162 | 126 - 150 | 118 - 157 |
